# An update on the clinical evidence that supports biosimilar approvals in Europe

**DOI:** 10.1111/bcp.13586

**Published:** 2018-04-27

**Authors:** Johanna Mielke, Bernd Jilma, Byron Jones, Franz Koenig

**Affiliations:** ^1^ Statistical Methodology Novartis Pharma AG 4056 Basel Switzerland; ^2^ Department of Clinical Pharmacology Medical University of Vienna Waehringer Guertel 18‐20 1090 Vienna Austria; ^3^ Center for Medical Statistics, Informatics and Intelligent Systems Medical University of Vienna Spitalgasse 23 1090 Vienna Austria

**Keywords:** clinical trials, drug development, statistics and study design, systematic review

## Abstract

**Aim:**

Sponsors and regulators have more than 10 years of experience with the development of biosimilars in Europe. However, the regulatory pathway is still evolving. The present article provides an update on biosimilar development in practice by reviewing the clinical development programmes of recently approved biosimilars in Europe.

**Methods:**

We used the European public assessment reports (EPARs) which are published by the European Medicines Agency (EMA) for a comparison of the clinical development programmes of the 37 approved biosimilars in Europe. Here, we present novel strategies in the development of biosimilars by focusing specifically on the 17 biosimilars that have gained approval in the last year, but we also compare additional key characteristics for all approved biosimilars.

**Results:**

The high variability of the clinical development strategies that we found previously was confirmed in the present analysis. Compared with earlier biosimilar applications, more nonstandard development strategies have been used recently. This includes, for example, applications without any studies in patients, and more complex study designs. During this study, we found that the EPARs for biosimilars seem to be improving; however, we identified important details which were still often missing. We provide a proposal for a checklist of the minimum information that should be included in biosimilar EPARs for giving the general public insights into the rationale for the approval of biosimilars.

**Conclusions:**

European regulators still seem to be open to consider approaches that differ from the guidelines or previous applications, as long as justification is provided.

## Introduction

As with a generic drug, a biosimilar is developed and approved as a copy of an already marketed product (the reference product) that has lost its patent protection. However, as the reference product for a biosimilar is a biological molecule (‘biologic’) instead of a small‐molecule drug, even though the main concept of biosimilars and generics is comparable, there are some fundamental differences: biosimilars are much more complex and they are produced in living cells 1. This makes the product sensitive to small changes in the production environment, so that even for the originator company that possesses most knowledge about the reference product, it would not be possible to produce an exact copy. This is why biosimilars only need to be ‘similar’ and not identical to the reference product 2. In addition, most biological drugs are so complex that they cannot be fully characterized by their chemical structure 1. Obviously, this is a complication not only for their development, but also for the showing of similarity. In fact, the abbreviated development programme that is used for the approval of generics is not adequate for biosimilars, and more evidence is required 3.

It is the responsibility of the sponsor of the biosimilar to convince the regulatory agencies that ‘any differences between it and its reference medicine will have been shown not to affect safety or effectiveness’ 4. In order to show that the proposed product has this property, sponsors have to submit the results of a comprehensive comparability exercise on quality, and nonclinical and clinical properties to regulatory authorities 2. Our work is focused on clinical development in Europe. For information on clinical biosimilar development in practice in other highly regulated markets, we refer the reader to, for example, Arato 5 for Japan or Hung *et al*. 6 for the US.

Previously, we investigated how the guidelines provided by the European Medicines Agency (EMA) are put into practice by conducting a systematic review of the clinical development programmes of the 21 biosimilars that had been approved in Europe at the time of acceptance of our first systematic review 7. We found that approval was possible even in cases in which the guidelines were not followed or not all primary endpoints were met. We also found that the variation between the clinical development strategies was high. Interestingly, this variability was also observed within an active substance, indicating that sponsors have some flexibility on the strategy they adopt for biosimilar development. However, many of the development programmes that we analyzed started at a point in time at which experience with biosimilars in practice was limited. One might wonder if more recently approved biosimilars follow a more standardized approach or if the same variability and flexibility is still present. In the study presented in this paper, we examined recent developments in clinical biosimilar development by looking at novel trends in the planning and analysis of the clinical trials of the 17 biosimilars that had been approved since the acceptance of our previous systematic review in August 2016 7. This included, for example, innovative study designs and development programmes without any studies in patients. In addition, we compared additional key characteristics of all successful biosimilar programmes. We focused on the choice of equivalence margins, the study population and the sample size calculation. In all these analyses, we compared biosimilars with each other, and biosimilar development in practice with regulatory requirements.

## Methods

The main source for our analyses was the so‐called European public assessment reports (EPARs) that were published by the EMA and are available online 8, 9, 10, 11, 12, 13, 14, 15, 16, 17, 18, 19, 20, 21, 22, 23, 24, 25, 26, 27, 28, 29, 30, 31, 32, 33, 34, 35, 36, 37, 38, 39, 40, 41, 42, 43, 44. If essential information was missing in the EPARs, we additionally conducted an online search for the missing information using databases such as PubMed, Isi Web of Science, clinicaltrials.gov, EudraCT and Google Scholar, with key words such as the drug name and the sponsor, the international nonproprietary name and the sponsor or the trial identifier of the sponsor. However, if not stated otherwise, all information presented here was taken from the EPARs.

For deciding if the recommendations provided by the EMA were followed, we compared the information provided in the EPARs with the published guidelines. The EMA distinguishes between overarching guidelines 2, 45, 46, which give very general advice, and product‐specific guidelines, which are focused on a specific group of treatments only and give detailed information – e.g. on proposed study designs and recommended endpoints. These guidelines are not a legally binding regulatory instrument but may be overruled by available data and a rational justification of why a guideline was not followed in a specific case. So far, eight different product‐specific guidelines have been published 47, 48, 49, 50, 51, 52, 53, 54.

As in our previous publication 7, we focused on approved biosimilars only. Products that had been withdrawn prior to the decision of the Committee for Medicinal Products for Human Use (CHMP), and the two products for which the CHMP adopted a negative opinion (Solumarv, Alpheon) were not analyzed. In addition, products that had been withdrawn after market authorization (e.g. Biograstim 55) were not in our focus of interest.

It is important to note that not all 37 biosimilars are in fact different products. Sometimes, companies jointly develop a biosimilar but market the product separately, or companies market identical products under different brand names in order to ensure that the approved biosimilar complies with the different local regulations in the member states of the European Union. In this case, the submitted clinical studies are identical, and therefore we did not consider the applications separately. In this article, this is indicated with a slash (e.g. Blitzima/Ritemvia/Rituzena/Truxima).

### Nomenclature of targets and ligands

Key protein ligands in this article are hyperlinked to corresponding entries in http://www.guidetopharmacology.org, the common portal for data from the IUPHAR/BPS Guide to PHARMACOLOGY 56.

## Results

**Figure 1:**
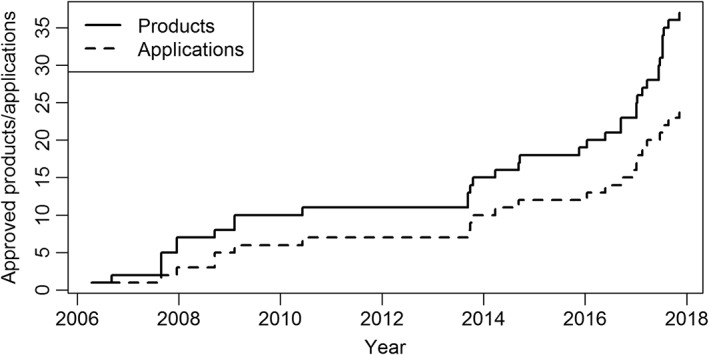
Biosimilar approvals over time. The dotted line gives the number of applications and the solid line indicates the number of products. In the case of different approval dates for products within one application, the earlier date is used

While in the past, only a few biosimilars gained approval per year and there were even years with no new biosimilar approvals, the number of approvals has increased greatly recently (see Figure 1). At present, a total of 37 biosimilars (23 different applications) for 12 active substances, have been approved in Europe, compared with 21 biosimilars (13 different applications) for seven active substances at the time of acceptance of our previous systematic review in August 2016 7. One of the biosimilars discussed in the previous review has been withdrawn (Biograstim 55). Therefore, we analyzed a total of 17 new biosimilars. Table 1 gives an overview of the new active substances; these included biosimilars for two new active anti‐inflammatory substances (tumour necrosis factor alpha blockers) and two new endocrinologically active substances. In addition, the first biosimilar of low‐molecular‐weight heparin for anticoagulant therapy gained approval during the period of study.

**Table 1:** Overview of biologics for which a biosimilar was authorized for the first time since August 2016

Active substance	Originator drug name	Originator company	Mechanism of action
**Endocrinologically acting drugs**			
**Insulin lispro**	Humalog	Eli Lilly	The active substance, insulin lispro, is a form of insulin which is absorbed more quickly by the body than human regular insulin, and can therefore act faster. It helps to control blood glucose levels, thereby alleviating symptoms and reducing the risk of complications of diabetes
**Teriparatide**	Forsteo	Eli Lilly	The active substance, teriparatide, is identical to part of the human parathyroid hormone. It acts like the hormone which stimulates bone formation by acting on osteoblasts (bone‐forming cells). It also increases the absorption of calcium from food and prevents too much calcium from being lost in the urine
**Anti‐inflammatory blockers of tumour necrosis factor alpha**			
**Rituximab**	MabThera	Roche	The active substance, rituximab, is a monoclonal antibody designed to recognize and attach to a protein called CD20, present on the surface of B‐lymphocytes. When rituximab attaches to CD20, it causes the death of B‐lymphocytes, which helps in lymphoma and chronic lymphocytic leukaemia (where B‐lymphocytes have become cancerous) and in rheumatoid arthritis (where B‐lymphocytes are involved in joint inflammation). In granulomatosis with polyangiitis (GPA) and microscopic polyangiitis (MPA), destroying the B‐lymphocytes lowers the production of antibodies thought to play an important role in attacking the blood vessels and causing inflammation
**Adalimumab**	Humira	AbbVie	The active substance, adalimumab, is a monoclonal antibody (a type of protein) that has been designed to recognize and attach to a substance in the body called tumour necrosis factor (TNF). This substance is involved in causing inflammation and is found at high levels in patients with the diseases that adalimumab is used to treat. By attaching to TNF, adalimumab blocks its activity, thereby reducing inflammation and other symptoms of the diseases
**Anticoagulants**			
**Enoxaparin sodium**	Clexane	Sanofi‐Aventis	In the *in vitro* purified system, enoxaparin sodium has high anti‐factor Xa activity (approximately 100 IU mg^–1^) and low anti‐factor IIa or anti‐thrombin activity (approximately 28 IU mg^–1^) , with a ratio of 3.6. These anticoagulant activities are mediated through anti‐thrombin III, resulting in anti‐thrombotic activity in humans

The information for biologics for which a biosimilar was approved earlier can be found in 7. The mechanism of action is quoted with only minor modifications from the ‘EPAR ‐ Summaries for the public’ available in 57 or from https://www.medicines.org

### Product‐specific guidelines and their implementation in practice

The required amount of information that is necessary to convince regulators clearly depends on the complexity of the molecule, the availability of established biomarkers and the sensitivity of clinical endpoints 45. Therefore, giving general recommendations on the amount of required evidence is not possible, and the variety of the reference products leads to very different strategies for biosimilar development 7. In order to address the more specific requirements of particular drug classes, the EMA publishes product‐specific guidelines. However, it is important to note that, in most cases, these guidelines are not developed prior to the start of development of the first biosimilar of a specific class. This is shown in Table 2; in the case of only two drug classes (low‐molecular‐weight heparins and insulins) was the first guideline available at least 3 years prior to approval of the first biosimilar. For the recently approved first biosimilar containing the active substance http://www.guidetopharmacology.org/GRAC/LigandDisplayForward?ligandId=4448, there has still been no product‐specific guideline published. By contrast, a guideline on products containing interferon beta is available, but no biosimilar has yet been approved in this class.

**Table 2:** Comparison of the publication date of the first product‐specific guideline and the date of application and the approval date of the biosimilar within the product class

Guideline (first publication)	Active substance	Biosimilar (application date/approval date)
**Recombinant erythropoietins (22.03.2006)**	Epoetin zeta	Silapo/Retacrit (−/18.12.2007)
	Epoetin alfa	Epoetin Alfa Hexal/Abseamed/Binocrit(−/28.08.2007)
**Recombinant granulocyte colony‐stimulating factor (22.02.2006)**	Filgrastim	Zarzio/Filgrastim Hexal (06.09.2007/06.02.2009)Tevagrastim/Ratiograstim (29.01.2007/15.09.2008)Nivestim (27.02.2009/08.06.2010)Grastofil/Accofil^a^ (30.04.2012/18.10.2013; 24.03.2014/18.09.2014)
**Recombinant follicle‐stimulating hormone (06.03.2013)**	Follitropin alfa	Ovaleap (28.02.2012/27.09.2013)Bemfola (30.10.2012/27.03.2014)
**Recombinant human insulin and insulin analogues (22.02.2006)**	Insulin glargine	Abasaglar (03.06.2013/09.09.2014)Lusduna (04.12.2015/04.01.2017)
	Insulin lispro	Insulin lispro Sanofi (07.09.2016/19.07.2017)
**Somatropin (22.02.2006)**	Somatropin	Omnitrope (−/12.04.2006)
	Teriparatide	Terrosa/Movymia^a^ (27.11.2015/04.01.2017; 30.11.2015/11.01.2017)
**Monoclonal antibodies (15.06.2012)**	Etanercept	Benepali (03.12.2014/14.01.2016)Erelzi (11.11.2015/23.06.2017)
	Infliximab	Remsima/Inflectra^a^ (01.03.2012; 26.06.2012/10.09.2013)Flixabi (03.03.2015/26.05.2016)
	Rituximab	Rixathon/Riximyo^a^ (11.04.2016; 09.12.2016/15.06.2017)Blitzima/Ritemvia/Rituzena/Truxima^a^ (06.03.2017; 03.03.2017/13.07.2017; 09.10.2015/17.02.2017)
	Adalimumab	Amgevita/Solymbic (03.12.2015/22.03.2017)Imraldi (21.06.2016/24.08.2017)Cyltezo (27.10.2016/10.11.2017)
**Low‐molecular‐weight heparins (19.03.2009)**	Enoxaparin sodium	Inhixa/Thorinane^a^ (27.05.2015; 06.02.2015/15.09.2016)
**Interferon beta (06.03.2013)**		

a Different approval and/or submission date, but the submitted studies are identical; − date is not specified in the European public assessment report

Even in the case where product‐specific guidelines were available at the time of development, these were not necessarily followed. We identified cases in which companies provided more information than explicitly required and also a case in which it seemed that the sponsor had an opinion that was incompatible with the guidelines, and decided to pursue a different approach than recommended. It is important to acknowledge that the reason for apparent noncompliance with the guidelines in operation at the time of approval is in some cases explainable by the fact that the companies followed an earlier version of the guidelines which was valid at the time the development of the product started. This was taken into account in the following examples.

To illustrate the case in which apparently more evidence than required was provided, we use the currently adopted guideline on recombinant human http://www.guidetopharmacology.org/GRAC/LigandDisplayForward?ligandId=5012 and insulin analogues 49. In this guideline, it is stated that ‘demonstration of similar pharmacokinetic (PK) and pharmacodynamic (PD) profiles is considered the mainstay of proof of similar efficacy of the biosimilar and the reference insulin’. The guideline continues to state the following: for PK/PD analyses, the study population can consist of healthy volunteers or type 1 diabetes patients. Concerning efficacy and safety studies, it is clearly stated that ‘there is no anticipated need for specific efficacy studies since endpoints used in such studies, usually HbA1c, are not considered sensitive enough to detect potentially clinically relevant differences between two insulins’. Safety studies should focus on immunogenicity assessment and use type 1 diabetes patients. In addition, it is stated that ‘in certain cases, a pre‐licensing safety study including immunogenicity assessment may be waived’. It should be noted that the idea of using PK/PD data as the pivotal piece of evidence is in line with the philosophy of the Hatch–Waxman Act 1984, in which it was acknowledged that evidence obtained during PK/PD assessment can be highly relevant for showing therapeutic equivalence between two products. This was discussed by Warren 58 in the context of biosimilar development. In summary, it is recommended that emphasis be put on the PK/PD phase of development.

To date, three biosimilars containing insulin have been approved; Abasaglar 24 and Lusduna 31 are biosimilars with the active substance insulin glargine, and Insulin lispro Sanofi 42 is a biosimilar with the active substance insulin lispro. The revised and above‐mentioned product‐specific guideline was published in 2015 and was therefore not available during the development of Abasaglar (approved in 2014). However, the first version of the guideline 59 was published in 2006 and the recommendation concerning the need for efficacy comparisons, even though much less detailed than in the latest version, is comparable. Therefore, we can assume that the expectations outlined above were commonly known for all sponsors of approved biosimilar insulins. Table 3 shows the study population, sample size and number of studies for the three biosimilars. All three sponsors submitted large efficacy and safety studies although this was not required in the guideline. Efficacy and extensive safety and immunogenicity data are presented. The rationale for the inclusion of diabetes type 2 patients in phase III trials is especially unclear because efficacy studies are, in general, not required for this active substance and, according to the product‐specific guideline, it is recommended that diabetes type 1 patients are included for safety and immunogenicity assessments. Overall, the option to conduct a development programme with limited clinical data, as proposed in the product‐specific guideline, was not used. In the EPARs, it is not explained why the applicants provided the extensive safety and efficacy comparisons. However, it is clearly stated that these studies were not required from a regulatory point of view. For example, in the EPAR of Insulin lispro Sanofi, it is stated that ‘as these studies are not formal requirements according to the CHMP Guideline on similar medicinal products containing recombinant human insulin, they are only considered as supportive for efficacy’ 42. Therefore, one can speculate why companies conduct these additional studies. One possible reason might be that they conduct global development programmes and intend also to submit the data package to other regulatory authorities which might require more extensive clinical trials. Another reason might be that they decided on a low‐risk approach by conducting more studies than explicitly required to avoid delays during the approval procedure due to discussions with regulators. However, as we only use publicly available information, it is not possible to substantiate any of these presumptions.

**Table 3:** Study population, sample size and number of studies for biosimilars containing insulin 24, 31, 42

Product	PK/PD			Efficacy/safety		
	Study population	Sample size	Number of studies	Study population	Sample size	Number of studies
**Abasaglar**	Healthy volunteers, type 1 diabetes	231	5	Type 1 diabetes, type 2 diabetes	1295	2
**Lusduna**	Healthy volunteers, type 1 diabetes	255	4	Type 1 diabetes, type 2 diabetes	1030	2
**Insulin lispro Sanofi**	type 1 diabetes	30	1	Type 1 diabetes, type 2 diabetes	1039	3

Studies in which the biosimilar is not included (e.g. comparison of the US with the EU reference product) are not listed. PD, pharmacodynamic; PK, pharmacokinetic

An example for the opposite strategy is the application for Inhixa/Thorinane (http://www.guidetopharmacology.org/GRAC/LigandDisplayForward?ligandId=6811 sodium). The product‐specific guideline, which was published in 2009 60, was in operation during the time of development and states that PK studies cannot be performed; instead, PD parameters [anti‐factor Xa (anti‐FXa), anti‐factor IIa (anti‐FIIa)] should be compared using a single‐dose crossover study design. If the product is licensed for the intravenous (IV) or intra‐arterial route, not only the subcutaneous, but also the IV route of administration must be used. In addition, it is stated that ‘a clear correlation between surrogate PD parameters (anti FXa or anti FIIa) and clinical outcome has not been established’ and that is why at least one parallel‐group trial for demonstrating equivalence in efficacy and safety is required. Comparing the clinical development programme of the sponsor 28, 29 with the guideline, we observe a clear mismatch: no phase III studies were conducted, which were originally required, and only 20 healthy volunteers were included in a PD study. No IV route of administration was used. Secondary endpoints failed, but the sponsor argued that the study was not powered to show equivalence on secondary endpoints. It is common practice not to power studies for success on secondary endpoints; however, we note that in general, multiplicity often is not considered in biosimilar development 61. In total, the development programme of the sponsor contradicts the guideline in most points, but the application was successful nonetheless.

Inhixa/Thorinane was approved on 15 September 2016. In November 2016, the CHMP published a revised product‐specific guideline 48. This guideline reflects the development programme of the sponsor. In the EPAR 28, 29, it is stated that ‘during the CHMP Scientific Advice (SA) procedures, the applicant claimed that PK/PD parameters such as anti‐Xa, anti‐IIa and TFPI [tissue factor pathway inhibitor] activities are more sensitive to detect potential differences in efficacy than clinical equivalence. This was endorsed by the CHMP’.

Whether 20 healthy volunteers can provide enough evidence that a biosimilar has comparable efficacy and safety with the reference product is controversial. Imberti *et al*. 62 state that ‘the authorizative path adopted by EMA for the introduction of biosimilar LMWHs [low‐molecular‐weight heparins] in Europe raises in our opinion some relevant concerns regarding efficacy and safety of these drugs’. In addition, they argue that ‘even stronger concerns are raised by the conclusions about safety, which are based just on a small‐sized PK/PD study in healthy volunteers’. Overall, they ‘advise the Italian National Health Authorities not to entrust safety assessment to the post‐marketing surveillance only, but to promote well designed and powered studies’. The lack of data for a firm conclusion on safety is also acknowledged in the EPAR 28, 29. For example, it is stated that ‘the presented clinical safety data derived from a comparative PK/PD study were too scarce to conclude on a comparable safety profile of test and reference medicinal products’. According to the EPAR 28, 29, the sponsor also at first did not present ‘a strategy of *in vitro* and/or *in vivo* assays to allow for waiving of clinical safety data’, but provided additional analysis during the application procedure, and comparative *in vitro* studies ‘were able to diminish immunogenicity concerns’. It is also important to note that the main safety concern is heparin‐induced thrombocytopenia and thrombosis (HITT). This is a very rare event and therefore difficult to assess in a limited patient population. Thus, it was concluded in the EPAR that ‘due to the low incidence of HITT, the conduct of a comparative clinical safety study was considered insensitive and unfeasible’ 28, 29. Therefore, in light of the totality of the data, it was concluded that the provided evidence was sufficient for granting market authorization.

Overall, this example shows that, in the case where a sponsor has a strong scientific rationale for a specific development programme, regulators in Europe still seem to be open minded to alternative development strategies, even for cases where a product‐specific guideline has already been issued by the EMA. The example also shows the value of a sophisticated analysis at the quality level; in the EPAR for Inhixa, it is stated explicitly in the conclusion 28, 29 that ‘in light of established biosimilarity on quality level, the remaining uncertainty that the safety profile of Inhixa and Clexane [the originator] differs significantly was considered low enough to conclude on similarity’. Therefore, it seems to be possible to push more weight to the quality part of the assessment if desired. In this respect, the EMA has published a draft reflection paper on statistical issues related to quality assessment 63. This also demonstrates that, in the future, quality data will become even more relevant for the approval of biosimilars.

It is important to note that, although Inhixa/Thorinane was the first product that gained approval without conducting efficacy/safety studies in patients, there exists now a second approved biosimilar with a very limited clinical data package: Movymia/Terrosa (teriparatide). So far, no product‐specific guideline is available for teriparatide. The sponsor based the decision for clinical equivalence on showing comparable PK profiles in 54 healthy volunteers 30, 32. Initially, the sponsor did not intend to submit any additional PD, efficacy or safety data. According to the EPAR, regulators requested information on the comparability of PD parameters, and the sponsor provided measurements which were collected during the PK study (PD marker: serum calcium). As there was some uncertainty regarding safety and immunogenicity, the sponsor agreed to provide data from a phase III study that is to be undertaken in Japan post‐marketing. According to the EPAR, it is expected that these data will be available in 2018 or 2019.

### Choice of study population

Biologics are often approved for various indications. Obviously, the goal of companies that are developing biosimilars is to get approval for all indications of the reference product. During development, not all indications are studied, but trials in selected indications are conducted. The other indications are granted using the concept of extrapolation 64. In practice, this approach has been used in all applications for biosimilars so far, and in most cases all indications of the reference product have been granted for the biosimilars 7. As only selected indications are studied, the choice of the study population is crucial. In the overarching guideline 45, it is stated that the ‘study population should generally be representative of approved therapeutic indication(s) and be sensitive for detecting potential differences between the biosimilar and the reference’ product. Table 4 shows the patient populations studied in the efficacy and safety studies. For eight active substances, at least two applications were successful and phase III studies for these were conducted. In only five out of these eight cases was the patient population identical. For the remaining three active substances, in one case the study populations were different, and in the other two at least one sponsor decided to study additional indications.

**Table 4:** Study population and sample sizes for phase III studies 8, 9, 10, 11, 12, 13, 14, 15, 16, 17, 18, 19, 20, 21, 22, 23, 24, 25, 26, 27, 28, 29, 30, 31, 32, 33, 34, 35, 36, 37, 38, 39, 40, 41, 42, 43, 44

Active substance	Biosimilar	Study population	Sample size
**Epoetin Alfa/Zeta**	Silapo/Retacrit	Haemodialysis patients with renal anaemia	609
		Haemodialysis patients with end‐stage renal failure and renal anaemia	402
		Cancer patients with chemotherapy‐induced anaemia	216
	Epoetin Alfa Hexal/Abseamed/Binocrit	Chronic renal failure patients on haemodialysis	478
		Patients with cancer chemotherapy‐associated anaemia	114
**Filgrastim**	Zarzio/Filgrastim Hexal	Breast cancer patients	170
	Tevagrastim/Ratiograstim	Breast cancer patients	348
		Lung cancer patients	237
		Non‐Hodgkin lymphoma	92
	Nivestim	Breast cancer patients	279
	Grastofil/Accofil	Breast cancer patients	120
**Follitropin alfa**	Ovaleap	Infertile ovulatory women undergoing ART	299
	Bemfola	Infertile ovulatory women undergoing ART	273
**Insulin glargine**	Abasaglar	Type 1 diabetes mellitus	536
		Type 2 diabetes mellitus	759
	Lusduna	Type 1 diabetes mellitus	502
		Type 2 diabetes mellitus	528
**Insulin lispro**	Insulin lispro Sanofi	Type 1 diabetes mellitus	507
		Type 2 diabetes mellitus	505
		Type 1 diabetes mellitus on continuous insulin infusion	27
**Somatropin**	Omnitrope	Growth hormone deficient patients	89
		Growth hormone deficient patients	51
**Teriparatide**	Movymia/Terrosa	X	X
**Etanercept**	Benepali	Rheumatoid arthritis	596
	Erelzi	Moderate to severe plaque psoriasis	531
**Infliximab**	Remsima/Inflectra	Rheumatoid arthritis	606
	Flixabi	Rheumatoid arthritis	584
**Rituximab**	Blitzima/Ritemvia/Rituzena/ Truxima	Rheumatoid arthritis	372
		Advanced follicular lymphoma patients	121
	Rixathon/Riximyo	Advanced follicular lymphoma patients	627
		Rheumatoid arthritis	173
**Adalimumab**	Amgevita/Solymbic	Moderate to severe plaque psoriasis	350
		Moderate to severe rheumatoid arthritis	526
	Imraldi	Rheumatoid arthritis	544
	Cyltezo	Moderate to severe rheumatoid arthritis	645
**Enoxaparin sodium**	Inhixa/Thorinane	X	X

ART, assisted reproduction techniques; X, no phase III study

An example of a development programme with completely different patient populations is that of the active substance http://www.guidetopharmacology.org/GRAC/LigandDisplayForward?ligandId=6789. Two biosimilars have been approved for this active substance: Benepali and Erelzi. The studies for Benepali were conducted in patients with rheumatoid arthritis, and for Erelzi in patients with plaque psoriasis. The product‐specific guideline on monoclonal antibodies 51 states only that ‘the most sensitive patient population and clinical endpoint is preferred to be able to detect product‐related differences’, but gives no specific recommendations. In the EPAR for Erelzi 38, it is stated that ‘the CHMP preferred rheumatoid arthritis (RA) to psoriasis as a model demonstrating equivalence, since patients with psoriasis may concern a more heterogeneous population, as a variety of prior treatments can be applied before the use of etanercept’. However, the sponsor was able to diminish doubts by conducting additional sensitivity analyses. As the sample size is comparable for both applications (Erelzi: 531; Benepali: 596), the burden on the sponsor might be similar. Therefore, it is not clear why the sponsor chose psoriasis as the studied indication in the application for Erelzi even though it was advised against and additional justification had to be provided. Again, this example confirms that sponsors have some flexibility in the set‐up of the clinical development programme.

### Innovative study designs and switchability assessment

Table 5 shows the details of the studies conducted for the biosimilars approved after August 2016. In the first systematic review 7, we reported that for PK/PD assessments, 2 × 2 crossover designs were used predominantly, and this is still one of the most frequently used study designs. However, in six out of the 10 new applications, comparisons with not only the EU, but also the US reference product are reported at the PK and/or PD level; in the previous report, this was the case for only three out of 13 applications. These studies might be part of global development programmes and serve as a bridge between the US and EU originator product, so that the large phase III trials only need to be conducted with either the US or the EU reference product 65. For the bridging studies, mostly three‐period crossover designs were used, but three‐arm parallel‐group designs were also conducted occasionally (e.g. for Flixabi).

**Table 5:** Overview of study designs used in the clinical development programme for getting approval as a biosimilar, for products approved from August 2016

Active substance	Product	Study design	*N*	Single/multiple dose	Route of administration, dose	PK	PD	E	S
**Insulin glargine**	Lusduna	2 × 2 crossover	24	Single	SC, 0.4 units kg^−1^	–	–	–	–
		3‐period crossover (EU reference *vs*. US reference *vs*. test)	109	Single	SC, 0.4 units kg^−1^	X	X	–	X
		2‐treatment, 4‐period crossover	76	Single	SC, 0.4 units kg^−1^	X	X	–	–
		2 × 2 crossover (viral *vs*. cartridge formulation of test product)	46	Single	SC, 0.4 units kg^−1^	–	–	–	–
		Parallel group^a^^,^*	502	Multiple	SC, previous dose	–	–	X	X
		Parallel group*	528	Multiple	SC, previous dose	–	–	X	X
**Insulin lispro**	Insulin lispro Sanofi	3‐period crossover (EU reference *vs*. US reference *vs*. test)	30	Single	SC, 0.3 units kg^−1^	X	X	–	X
		Parallel group (EU reference *vs*. US reference *vs*. test)^b^^,^*	507	Multiple	SC, n.s.	–	–	X	X
		Parallel group (EU reference *vs*. US reference *vs*. test)^c^^,^*	505	Multiple	SC, n.s.	–	–	X	X
		2 × 2 crossover*	27	Multiple	External pump for continuous SCII	–	–	–	X
**Teriparatide**	Movymia/Terrosa	2 × 2 crossover^l^	54	Single	SC, 20 μg/80 μl	X	X	–	X
**Etanercept**	Erelzi	2 × 2 crossover	108	Single	SC, 50 mg	X	–	–	X
		2 × 2 crossover (autoinjector *vs*. prefilled syringe of test product)	102	Single	SC, 50 mg	X	–	–	X
		2 × 2 crossover	108	Single	SC, 50 mg	X	–	–	X
		2 × 2 crossover (test *vs*. US)	114	Single	SC, 50 mg	–	–	–	X
		Parallel group/switching design^d^^,^ m ^,^*	531	Multiple	SC, 50 mg^n^	X	X	X	X
**Rituximab**	Blitzima/Ritemvia/ Rituzena/Truxima	Parallel group^o^	154	Multiple	IV, 1000 mg^p^	X	X	X	X
		Single‐arm	1	Multiple	IV, 375 mg m^−2^	–	–	–	–
		3‐arm parallel group (EU reference *vs*. US reference *vs*. test)^e^^,^*	372	Multiple	IV, 1000 mg^p^	X	X	X	X
		Parallel group^f^^,^*	121	Multiple	IV, 375 mg m^−2^ every three weeks	X	X	X	X
	Rixathon/Riximyo	Parallel group (test *vs*. Japanese reference)	6	Multiple	IV, 375 mg m^−2^ weekly	X	–	–	X
		Parallel group^g^^,^*	173	Multiple	IV, 1000 mg^p^	X	X	X	X
		Parallel group^h^^,^*	627	Multiple	IV, 375 mg m^–2,^ q	X	X	X	X
**Adalimumab**	Amgevita/Solymbic	3‐arm parallel group (EU reference *vs*. US reference *vs*. test)	203	Single	SC, 40 mg	X	–	–	X
		Parallel group^i^, ^r^ (test *vs*. EU)*	350	Multiple	SC, 80 mg week 1 day 1, 40 mg e.o.w. from week 2	X	–	X	X
		Parallel group^j^ (test *vs*. US)*	526	Multiple	SC, 40 mg e.o.w.	X	–	X	X
	Imraldi	3‐arm parallel group (EU reference *vs*. US reference *vs*. test)	189	Single	SC, 40 mg	X	–	–	X
		Parallel group^k^^,^ s ^,^*	544	Multiple	SC, 40 mg e.o.w.	X	–	X	X
	Cyltezo	3‐arm parallel group (EU reference *vs*. US reference *vs*. test)	193	Single	SC, 40 mg	X	–	–	X
		3‐arm parallel group (EU reference *vs*. US reference *vs*. test)	324	Single	SC, 40 mg	X	–	–	X
		Parallel group (autoinjector vs. prefilled syringe of test product)	66	Single	SC, 40 mg	X	–	–	X
		Single‐arm	77	Multiple	SC, 40 mg e.o.w.	–	–	–	X
		Parallel group (test *vs*. US)^s^^,^*	645	Multiple	SC, 40 mg e.o.w.	X	–	X	X
**Enoxaparin sodium**	Inhixa/Thorinane	2 × 2 crossover	20	Single	SC, 40 mg	–	X	–	X

The information for all biosimilars which were approved earlier can be found in 7. Only studies undertaken prior to market authorization are listed. Studies in which the biosimilar is not included (e.g. comparison of the US with the EU reference product) are not listed.

Studies with * are phase III‐studies. All information is taken from the European public assessment reports [28–44]. E, efficacy; e.o.w., every other week; IV, intravenous; *N*, number of subjects; n.s., not specified; PD, pharmacodynamic; PK, pharmacokinetic; S, safety; SC, subcutaneous; SCII, subcutaneous insulin infusion; X, data from the study was discussed in this part of the European public assessment reportEudraCT‐ID:

a 2011–003971–12,

b 2013–002945–12,

c 2014–002844–42,

d 2012–002011–26,

e 2013–004555–21,

f 2013–004493–96,

g 2010–021184–32,

h 2010–019522–13,

i 2013–000537–12,

j 2013–000525–31,

k 2013–005013–13 Further details:

l Two‐stage design with the possibility for stopping at interim

m Period 1: parallel groups, period 2: parallel groups or crossover (switching), period 3: parallel group

n First 12 weeks 50 mg twice weekly, afterwards 50 mg once weekly

o This study has an open‐label, single‐arm extension to evaluate long‐term efficacy and safety

p Up to two courses; each course consists of two single infusions, with a 2‐week interval between the infusions

q Combination phase: 8 cycles, administered every 21 days, maintenance phase: 8 cycles, administered every 3 months

r Patients were re‐randomized at week 16 to continue on reference or to switch to test

s Patients were re‐randomized at week 24 to continue on reference or to switch to test.

In addition to the more frequent inclusion of the US reference product, we also noted the use of adaptive designs (sample size re‐estimation and stopping at interim) in more recent applications. These designs have the advantage that the sample size is not fixed at the beginning of the trial and there is flexibility to adjust it when information about the nuisance parameters that drive the sample size becomes available during an interim analysis. This reduces the risk of misspecification of the sample size and allows a trial to finish earlier and enrol fewer subjects if justified 66. On the other hand, it is necessary to emphasize the drawbacks of adaptive designs. First, adaptive designs can be more difficult to handle in practice for operational reasons. Secondly, it is necessary to take the possibility of a design adaptation into account while planning the statistical analysis, so that the overall Type I error rate is controlled, and this might require more complex methods and complicate the analysis. In addition, all potential adaptations have to be prespecified 67, 68.

Examples of adaptive designs used in practice can be found in the EPARs for Movymia/Terrosa (teriparatide) 30, 32 (two‐stage design), Abasaglar (insulin glargine) 24 (blinded sample‐size re‐estimation) and Cyltezo (http://www.guidetopharmacology.org/GRAC/LigandDisplayForward?ligandId=4860) 44 (blinded sample‐size re‐estimation). All examples show that regulators in Europe are open to the use of adaptive methodology for adjusting the sample size during the trial 69.

Another new type of trial design involves switching designs – e.g. the EGALITY study for Erelzi 70. In that study, patients started in a parallel‐group design, but a subset of the subjects started to switch between the biosimilar and the reference product after completion of the first treatment period and the assessment of the primary efficacy endpoint. The EMA clearly states 4 that the decision as to whether a patient can switch between the biosimilar and its reference product is not made during the centralized EMA approval procedure, and lies generally with the member states, whose health authorities have diverse positions on this question 71. Therefore, it is likely that this switching study was conducted to fulfil the requirements for interchangeability in the US 72, or national health technology assessment and reimbursement bodies, or for marketing purposes, but was not required specifically for approval in the EU. Nonetheless, even though the EMA states that it will not give any opinion on switchability 4, this topic is discussed in some of the EPARs. For example, it is stated in the benefit‐risk assessment for Erelzi that ‘maintenance of efficacy was shown up to 52 weeks for continuous treatment of Erelzi and Enbrel [the originator] from baseline, as well as in the switching groups’ 38.

So far, the study design for Erelzi is the only one with multiple switches that is mentioned in an EPAR. However, single transitions from the reference product to the biosimilar are more common, most likely because of regulatory expectations in the US. While a study that incorporates multiple switches between the biosimilar and its originator (e.g. the study design of the EGALITY study) is expected to be required only for getting approval as an ‘interchangeable biosimilar’ 72, a descriptive assessment of the potential for increased immunogenicity caused by a single transition from the originator to the biosimilar might be required also for the approval as a ‘biosimilar’ in the US 73. Frequently, comments are made in the EPARs on the impact of these switches on the effect of the treatment. For example, for Imraldi (adalimumab) 43, it is stated that ‘the switch of the treatments to clarify the interchangeability between the biosimilar and originator is not a requirement within EU, but supports the Applicant's global program, and is as such acceptable’. By contrast, for Abasaglar (insulin glargine) 24, it is stated that ‘importantly, both studies provided data on patients switching from Lantus [the originator] to LY2963016 [Abasaglar] at the same dose regimen; no difference in dose changes after titration to tighten glucose blood control was reported between the two treatment arms’, suggesting that providing switching data is considered to be an important piece of evidence. It seems as if the evaluation of switchability depends on the rapporteur and is not yet consistent in EPARs.

### Equivalence vs. non‐inferiority assessment

In general, sponsors are asked to show equivalence and not non‐inferiority. So far, most companies have followed the recommendation and conducted equivalence studies.

However, non‐inferiority designs were used in the two applications for Abasaglar and Lusduna (both insulin glargine) and in the application for Blitzima/Ritemvia/Rituzena/Truxima (http://www.guidetopharmacology.org/GRAC/LigandDisplayForward?ligandId=6780). For Abasaglar, both efficacy studies used a non‐inferiority design. As described previously, efficacy studies are not required in the product‐specific guideline for insulins 49. That is why it is stated in the EPAR 24 that ‘given the supportive role of these phase III studies in the biosimilar programme, the statistical methodology for these studies does not raise major concerns’. For Lusduna 31, the primary objective of the study was non‐inferiority and the secondary objective was equivalence. In order to guarantee the Type I error rate (the patient's risk), a step‐down procedure was used – i.e. first the primary objective was tested (non‐inferiority) and only if this hypothesis was rejected was the secondary objective (equivalence) tested. In this example, both non‐inferiority and equivalence were shown. However, one might wonder how the EMA would have handled the application if equivalence had explicitly been tested but could not be shown.

For Blitzima/Ritemvia/Rituzena/Truxima, the smaller of the two efficacy studies used a non‐inferiority approach (see Table 5). No comment is given in the EPAR 33, 39, 40, 41 as to why a non‐inferiority design was considered acceptable. However, it is argued in the guideline 45 that ‘a non‐inferiority trial may only be accepted where the possibility of a significant and clinically relevant increase in efficacy can be excluded on scientific and mechanistic grounds’. Along these lines, even extremely low doses of rituximab (several hundred‐fold lower than those currently authorized) deplete almost all circulating CD20+ B‐cells in the circulation, at least temporarily 74. At higher doses, all B‐cells eventually also are eliminated from the tissues and the maximum PD effect is reached, which cannot be further increased (ceiling effect).

### Choice of margins in efficacy trials

As in all clinical development programmes, the statistical analysis should be prespecified. As biosimilar trials are equivalence (or non‐inferiority) trials, the equivalence margins are also crucial for the test decision and need to be prespecified. In efficacy trials, the studied indications are diverse and, as the margins have to reflect a difference that is not relevant from a clinical point of view and this acceptable difference depends on the disease, it is not possible to use a standardized margin, as it is done for bioequivalence studies 75. In general, the equivalence margins in efficacy trials must be clinically and statistically justified 45. This is comparable with the choice of margins in non‐inferiority trials 76. Table 6 gives an overview of the choice of margins in practice. It shows that the margins appear to not have been prespecified in only two cases. For Omnitrope (http://www.guidetopharmacology.org/GRAC/LigandDisplayForward?ligandId=4943), no margin is mentioned in the EPAR 8 at all (it might have been in the study protocol, but this is not publicly available). A 95% confidence interval was calculated and it is stated that this shows that the difference between the treatments is not clinically relevant. Therefore, it is unclear if a formal testing procedure was performed or a descriptive approach was used. The second case is the recently approved application for Blitzima/Ritemvia/Rituzena/Truxima (rituximab). In the EPAR 33, 39, 40, 41, it is stated that the main study for efficacy is a phase I study in patients with rheumatoid arthritis, with the internal code CT‐P10 1.1. The primary objective was PK equivalence and the study was powered only for this. Efficacy using the mean change from baseline in the 28‐joint Disease Activity Score (DAS28) 77 was evaluated and the equivalence margin was chosen *post hoc* and justified by historical data. The sponsor also provided a second study with the same efficacy endpoint as the primary endpoint, using the same equivalence margin. For the second study, this margin was prespecified according to the EPAR. From the EPAR, it is not clear why study CT‐P10 1.1 was presented as the pivotal efficacy study.

**Table 6:** Margins for efficacy assessment

Active substance	Biosimilar	*N*	Endpoint	Margin	Prespecified?	Justified?
**Epoetin Alfa/Zeta**	Silapo/Retacrit	609	Mean weekly dosage of epoetin per kg	(−14, 14)	Yes^a^	Yes, no details
	Epoetin Alfa Hexal/Abseamed/Binocrit	478	Mean absolute change in Hb level	(−0.5, 0.5)	Yes	X
**Filgrastim**	Zarzio/Filgrastim Hexal	Single‐arm study only				
	Tevagrastim/Ratiograstim	348	Duration of severe neutropenia during cycle 1	(−1, 1)	Yes	X
	Nivestim	279	Duration of severe neutropenia during cycle 1	(−1, 1)	Yes	X
	Grastofil/Accofil	Single‐arm study only				
**Follitropin alfa**	Ovaleap	299	Number of oocytes retrieved	(−3, 3)	Yes	Yes, no details
	Bemfola	273	Number of oocytes retrieved	(−2.9, 2.9)	Yes	Yes, references are given, but no details
**Insulin glargine**	Abasaglar	759	Change in HbA1c from baseline (percentage)	0.4% (non‐inferiority margin), if met 0.3%	Yes	Yes, not provided
	Lusduna	528	Change in HbA1c from baseline (percentage)	0.4% (non‐inferiority margin)	Yes	X
**Insulin lispro**	Insulin lispro Sanofi	507	Change in HbA1c from baseline (percentage)	0.3% (non‐inferiority margin)	Yes	X
**Somatropin**	Omnitrope	89	Height	X	X	X
**Teriparatide**	Movymia/Terrosa	Not studied				
**Etanercept**	Benepali	596	ACR20 responders	(−15, 15)	Yes	Yes, full information
	Erelzi	531	PASI75 responders	(−18, 18)	Yes	X
**Infliximab**	Remsima/Inflectra	606	ACR20 responders	(−15, 15)	Yes	Yes, statistical approach given, but no reference to historical information
	Flixabi	584	ACR20 responders	(−15, 15)	Yes	Yes, full information
**Rituximab**	Blitzima/Ritemvia/Rituzena/ Truxima	154	DAS28	(−0.6,0.6)	No	Yes (post‐hoc analysis), full information
	Rixathon/Riximyo	627	Overall response rate	(−12, 12)	Yes	Yes, full information
**Adalimumab**	Amgevita/Solymbic	526	ACR20 responders (risk ratio)	(0.738, 1.355)	Yes	Yes, reference is given, but no details
	Imraldi	544	ACR20 responders	(−15, 15)	Yes	Yes, full information
	Cyltezo	645	ACR20 responders at week 12	(−12, 15)	Yes	X
**Enoxaparin sodium**	Inhixa/Thorinane	Not studied				

If more than one (primary) endpoint was mentioned in the European public assessment report (EPAR), the endpoint listed first is provided as an example. If more than one study was provided, the pivotal study or (if that was not stated) the study with the larger sample size and comparison with the EU reference is reported. All information is taken from the EPARs 8, 9, 10, 11, 12, 13, 14, 15, 16, 17, 18, 19, 20, 21, 22, 23, 24, 25, 26, 27, 28, 29, 30, 31, 32, 33, 34, 35, 36, 37, 38, 39, 40, 41, 42, 43, 44. ACR20, subjects with at least 20% improvement according to the criterion of the American College of Rheumatology; DAS28, 28‐joint Disease Activity Score; Hb, haemoglobin; HbA1c, glycosylated haemoglobin; PASI75, subjects with a 75% improvement in the Psoriasis Area and Severity Index score; X, no information is given

a Endpoint was not successful; the applicant claimed that the EPAR from the reference product had been misread and argued to change the range to (−45, 45).

Even though margins were in most cases prespecified and stated in the EPAR, it is often unclear if these margins were clinically and statistically justified (see Table 6). In cases in which the margins were, according to the EPARs, justified, the justification is often not stated. Only for both biosimilars with the active substance rituximab, for Flixabi (http://www.guidetopharmacology.org/GRAC/LigandDisplayForward?ligandId=5004), Benepali (etanercept) and Imraldi (adalimumab), the provided information is sufficiently detailed that it would be possible for an external reader to replicate the derivations of the margins easily. The applications with full information on margin justification were all recently approved biosimilars, so one might hope that this information will be included more often in future EPARs.

As with other aspects of the development programme, the choice of margins can also be discussed and agreed with the EMA via their scientific advice (SA) procedure; but seeking SA is not mandatory and is at the discretion of the sponsor. Some sponsors might have sought SA beforehand, but others not. This might explain why sometimes the chosen margins were not considered acceptable in the assessment of the biosimilar applications. We note that the given SA is neither legally binding for the sponsor nor for the CHMP (for the later approval process). Therefore, the CHMP can also change its opinion on an agreed margin in the light of new data later on, or the sponsor might use a different margin. An example of a *post hoc* adjustment of the margins is in the application for Erelzi (etanercept), where the sponsor predefined an equivalence margin of (−18, 18) (see Table 6) for the difference in the responder rates according to the PASI75 criterion: a patient is classified as a PASI75 responder if the improvement from baseline in the Psoriasis Area and Severity Index (PASI) [78] is larger than 75%. In the EPAR 38, it is stated that ‘from a clinical perspective, the equivalence margins are considered too wide and not sufficiently justified, as these may include a relevant difference in effect size’. However, as the observed confidence intervals were very narrow and fell in the preferred range, which was (−10, 10), this was not considered to be an issue. While tightening the margins is not an issue from a statistical point of view for single endpoints 79, it is again unclear what the agency would have decided if the confidence interval had not fallen within the preferred, tighter margins. Studies are normally powered for a specific margin, and tightening the margins *post hoc* might lead to an underpowered study. Therefore, if the agency takes the liberty of tightening the equivalence margins, this might be considered as an unforeseeable risk for the sponsor. To mitigate this risk, it might be advisable for the sponsor to agree on the margin with regulatory authorities when planning the study – e.g. in the EU, by seeking SA from the EMA. Widening the margins *post hoc*, as it was done for Silapo/Retacrit 12, 13, potentially increases the Type I error risk (the patient's risk). Therefore, this should only be acceptable in cases in which a strong justification is provided.

### The enhanced quality of newer EPARs and suggestions for further improvement

We found the newer EPARs, in most cases, more structured and detailed compared with the EPARs of the first approved biosimilars (e.g. Omnitrope 8, Silapo/Retacrit 12, 13). For example, a summary table of the study results of the phase III trials is included in nearly all of the newer reports and that makes it easier to find information quickly. However, there are, from our point of view, still aspects that could be improved. First, all crucial information related to the set‐up of the studies should be included in the EPARs. This includes, e.g., a justification of the equivalence margins in phase III. Another example is information on the assumptions for the sample size calculation which is sometimes missing. In addition, the EudraCT‐ID, which allows additional information to be found on the trial in the EU Clinical Trials Register, is still often missing and in the few cases in which it is reported, it is often not correct (e.g. Movymia/Terrosa, Cyltezo, Inhixa/Thorinane). We also identified some inconsistencies between the EPARs (e.g. in the way that switchability assessments are judged). For an external reader, it is difficult to judge if there are scientific reasons for these differences (e.g. one product appeared to be ‘more’ switchable than the other) or if it depends on the specific person who wrote the report. For improving comparability, it would be useful to address the same aspects in all reports, especially in the ones with the same active substance. From a statistical point of view, it would be desirable to have all details of the innovative approaches stated. For example, for Cyltezo (adalimumab), a sample size re‐estimation was used 44, but no details or references about the approach were provided. This makes it difficult for sponsors to learn from the recently implemented novel methodology used by comparators. Indeed, in a different class of products, inconsistency and a lack of information in EPARs have already been reported 80. As with the CONSORT statement for reporting clinical trials 81, 82, analogous reporting guidelines for EPARs should be implemented. Barbui *et al*. 80 provided a proposal for a table for reporting the results of clinical trials in EPARs. However, as biosimilar trials have some specific characteristics, and the proposed table focused more on the reporting of the results and less on the planning of the study and interpretation of the results, we present a proposal for such a checklist, specific for EPARs for biosimilars, in Table 7. We also provide examples of good reporting practice to give concrete recommendations on the presentation of the development programmes.

**Table 7:** Some recommended discussion points and information that should be given in all European public assessment reports related to clinical biosimilar development

	Aspect	Minimal details	**Positive reporting example**
**General information**	Rapporteur and co‐rapporteur		Inhixa/Thorinane
	Scientific advice and protocol assistance procedures	Details on the scientific advices or protocol assistance procedures	Erelzi
	Details on time line of application procedure		Erelzi
**Study planning**	Study design	Type of design Exact treatment sequences Randomization, allocation ratios to sequences In case not a standard is design is used: rationale for the choice of design	Study XM02–02‐INT (Tevagrastim/Ratiograstim)
	Sample size	Number of subjects Details on sample size calculations, including explanations on assumptions for nuisance parameters Information on randomization Dealing with multiplicity (if applicable)	Full information on sample size calculation in study ABEB (Abasaglar)
	Study population	Rationale for choice of study population Inclusion/exclusion criteria	Table with inclusion/exclusion criteria for study CT‐P13 3.1 (Remsima/Inflectra)
	Treatment	Dose, dosage regimen, route of administration	Complete dosing information for study EP06–102 (Zarzio/Filgrastim Hexal)
	Endpoint	Classification into primary, secondary and exploratory endpoints	Listing of endpoints in study RGB‐10‐001 (Movymia/Terrosa)
	Margins	Margins and the justification of the margins including references to historical data if used Were the margins prespecified? If a non‐inferiority design was used: scientific arguments why this gives the same information as an equivalence design	Justification of margins for study SB2‐G31‐RA (Flixabi)
	Significance level	Significance level Was this level prespecified?	Study RGB‐10‐001 (Movymia/Terrosa)
	Importance of the study	If multiple studies were performed for achieving the same goal, which study is the pivotal study (if any)? What is the weight of the study for the development programme in the context of ‘totality of the evidence’? Was this weight prespecified?	Lusduna: clear indication that efficacy studies are supportive only
	Analysis method	In general: give enough details, such that a replication of the analysis is possible In case of adaptive designs/sample size re‐estimation: details on the analysis performed, including references to methodological papers	Phase III study for Ovaleap: statistical model is mentioned and the considered covariates are listed
	Analysis population	Definition of the analysis population (e.g. per protocol, full analysis set) and safety population	Study GCF071 (Nivestim)
	Multiplicity	Discuss if and how multiplicity was addressed when planning and analysing the results	Study P002 for Lusduna: it is clearly stated that no multiplicity adjustment is necessary because all endpoints have to be successful
	Formal aspects	EudraCT‐ID	Study SB5‐G31‐RA (Imraldi)
**Reporting of results**	Sample size	Flow of the study population	Flow chart for study XM17–05 (Ovaleap)
	Baseline characteristic	Demographic information Information on pretreatment (if applicable)	Study EGALITY (Erelzi)
	Primary endpoint	Point estimator (continuous endpoint) or number of responders (binary endpoint) Variance, intrasubject variability and intersubject variability (if applicable) Coefficient of variation (in PK/PD studies) Confidence interval and confidence interval level or *P*‐value	Study XM17–05 (Ovaleap)
	Additional justification	In the case of unexpected results (e.g. primary endpoint was missed): explanation, rationale for why the finding does not preclude biosimilarity and statement about the type of additional analyses and/or new studies that were conducted	Detailed explanations and clear description in the discussion on clinical pharmacology for Movymia/Terrosa
**Conclusion**	PK/PD	Equivalence in PK and PD demonstrated?	Movymia/Terrosa
	Efficacy and safety	Efficacy: comparable efficacy? Immunogenicity: equivalent or better immunogenicity profile for the biosimilar?* Safety: equivalent or better safety profile for the biosimilar?* Switchability (if applicable): switchability demonstrated?*	Benepali
	Residual uncertainty	Is there any residual uncertainty? If yes, how will this uncertainty be dealt with?	Movymia/Terrosa: clear statement of the residual uncertainty (immunogenicity) and the way that this uncertainty will be addressed (postmarketing data from Japan)
	Extrapolation	Rational for extrapolation	Imraldi

PD, pharmacodynamic; PK, pharmacokinetic

* A decision is necessary concerning whether or not these points are to be included in the EPAR. If it is included, it should be consistent in all EPARs.

## Discussion

The biosimilar landscape in Europe has widened considerably over the past year with 37 biosimilars now approved (previously: 21 up to August 2016) for 12 (previously: 7) active substances. The present literature review provided an update of biosimilar development in practice based on EPARs published by the EMA. We confirmed that the high variability of the submitted applications that had been reported previously 7 is still present by analyzing the study populations, sample sizes and equivalence margins for efficacy trials. Importantly, this variability was observed both for active substances for which the first biosimilar had been approved recently (e.g. rituximab) and also for biosimilars long since approved, for which a second application has been successful more recently (e.g. insulin glargine). Therefore, it seems as if the EMA is still open to considering alternative and innovative approaches.

An important question in biosimilar development over the past few years has been whether extensive clinical trials are necessary or if biosimilars could gain approval using quality data as the pivotal piece of evidence 83. In the overarching guideline, the EMA states that ‘in specific circumstances, a confirmatory clinical trial may not be necessary’ 2. This option was put into practice in two applications that were approved without studying efficacy, safety or immunogenicity in patients. By contrast, we found examples where the sponsors provided more information than explicitly required by the product‐specific guidelines. It has already been reported that the size of the company affects clinical trial programmes and their success 84. Comparing the companies involved in the applications without any studies in patients, using the Scrip 100 ranking of pharmaceutical companies by sales 85, with those which apparently provided more information than required, we noted that the latter companies are ranked as third (Sanofi), fifth (Merck) and 13th (Eli Lilly), whereas the former are ranked 41 (Stada) and 64 (Gedeon Richter) or are not in the top 100 companies at all (Techdow, Pharmathen). Even though these few examples are not sufficient to reach a definitive conclusion, they suggest that big pharma companies might be less willing to take the risk of delaying an approval by not providing enough evidence. Alternatively, one could speculate whether big pharma companies are hoping to improve their marketing activities by enabling their representatives to refer to large clinical trials. With more biosimilars gaining approval in the next few years, it will be possible to investigate this question more thoroughly.

As in our previous analysis, the present study used publicly available information only. We have no insights into the rationale behind the decisions of sponsors or regulators if these are not stated in the EPAR. That is why we cannot state the reasons for specific choices during development. However, our work shows the level of transparency that is achieved by the EMA in decision making during the assessment of biosimilar applications, and indicates the areas in which more information in EPARs is needed to be more comprehensible. We note that the EMA has started to release more documents as a result of its policy 0070 86. However, we recommend that all crucial information is included in the EPAR itself, to make it as easy as possible for the general public to access information.

## Conclusion

Regulators in Europe seem to be open to discuss alternative development strategies. This was observed in cases in which a biosimilar has already been approved and used, and also in cases in which a product‐specific guideline exists. Therefore, sponsors who would like to structure the development programme in a different way might have a fair chance of gaining approval in the end, if the alternative approach can be justified from a scientific point of view. For that purpose, early interactions with regulatory authorities, e.g. seeking SA from the EMA, are highly recommended.

## Competing Interests

There are no competing interests to declare.

*J.M. was supported by the Swiss State Secretariat for Education, Research and Innovation (SERI) under contract number 999754557. The opinions expressed and arguments employed herein do not necessarily reflect the official views of the Swiss Government. This project is part of the IDEAS European training network (http://www.ideas‐itn.eu/) from the European Union's Horizon 2020 research and innovation programme under the Marie Sklodowska‐Curie grant agreement No 633567. B.J. was supported by the FWF grant SFB54‐P04*.
